# Specific populations of urinary extracellular vesicles and proteins differentiate type 1 primary hyperoxaluria patients without and with nephrocalcinosis or kidney stones

**DOI:** 10.1186/s13023-020-01607-1

**Published:** 2020-11-11

**Authors:** Muthuvel Jayachandran, Stanislav V. Yuzhakov, Sanjay Kumar, Nicholas B. Larson, Felicity T. Enders, Dawn S. Milliner, Andrew D. Rule, John C. Lieske

**Affiliations:** 1 Division of Nephrology and Hypertension, College of Medicine and Science, Mayo Clinic, 200 First Street SW, Rochester, MN 55905 USA; 2 Division of Hematology Research, College of Medicine and Science, Mayo Clinic, 200 First Street SW, Rochester, MN 55905 USA; 3 Department of Physiology and Biomedical Engineering, College of Medicine and Science, Mayo Clinic, 200 First Street SW, Rochester, MN 55905 USA; 4 Biomedical Statistics and Bioinformatics, College of Medicine and Science, Mayo Clinic, 200 First Street SW, Rochester, MN 55905 USA; 5 Department of Laboratory Medicine and Pathology, College of Medicine and Science, Mayo Clinic, 200 First Street SW, Rochester, MN 55905 USA

**Keywords:** Microvesicles, Exosomes, Urinary vesicles, Urinary proteins, Oxalate, Renal calcification, Urinary stone disease

## Abstract

**Background:**

Primary hyperoxaluria type 1 (PH1) is associated with nephrocalcinosis (NC) and calcium oxalate (CaOx) kidney stones (KS). Populations of urinary extracellular vesicles (EVs) can reflect kidney pathology. The aim of this study was to determine whether urinary EVs carrying specific biomarkers and proteins differ among PH1 patients with NC, KS or with neither disease process.

**Methods:**

Mayo Clinic Rare Kidney Stone Consortium bio-banked cell-free urine from male and female PH1 patients without (n = 10) and with NC (n = 6) or KS (n = 9) and an eGFR > 40 mL/min/1.73 m^2^ were studied. Urinary EVs were quantified by digital flow cytometer and results expressed as EVs/ mg creatinine. Expressions of urinary proteins were measured by customized antibody array and results expressed as relative intensity. Data were analyzed by ANCOVA adjusting for sex, and biomarkers differences were considered statistically significant among groups at a false discovery rate threshold of Q < 0.20.

**Results:**

Total EVs and EVs from different types of glomerular and renal tubular cells (11/13 markers) were significantly (Q < 0.20) altered among PH1 patients without NC and KS, patients with NC or patients with KS alone. Three cellular adhesion/inflammatory (ICAM-1, MCP-1, and tissue factor) markers carrying EVs were statistically (Q < 0.20) different between PH1 patients groups. Three renal injury (β2-microglobulin, laminin α5, and NGAL) marker-positive urinary EVs out of 5 marker assayed were statistically (Q < 0.20) different among PH1 patients without and with NC or KS. The number of immune/inflammatory cell-derived (8 different cell markers positive) EVs were statistically (Q < 0.20) different between PH1 patients groups. EV generation markers (ANO4 and HIP1) and renal calcium/phosphate regulation or calcifying matrixvesicles markers (klotho, PiT1/2) were also statistically (Q < 0.20) different between PH1 patients groups. Only 13 (CD14, CD40, CFVII, CRP, E-cadherin, EGFR, endoglin, fetuin A, MCP-1, neprilysin, OPN, OPGN, and PDGFRβ) out of 40 proteins were significantly (Q < 0.20) different between PH1 patients without and with NC or KS.

**Conclusions:**

These results imply activation of distinct renal tubular and interstitial cell populations and processes associated with KS and NC, and suggest specific populations of urinary EVs and proteins are potential biomarkers to assess the pathogenic mechanisms between KS versus NC among PH1 patients.

## Background

Primary hyperoxaluria type 1 (PH1) is a rare metabolic disorder with significant morbidity and mortality. PH1 is caused by mutations of glyoxylate alanine aminotransferase (AGT), a hepatocyte peroxisomal enzyme responsible for conversion of glyoxylate to glycine, resulting in overproduction of oxalate [1, 2]. Age of clinical presentation varies from infancy to adulthood (median 5.5 years [3]) with 20 to 50% of patients having advanced chronic kidney disease (CKD) at the time of diagnosis, and end stage kidney disease (ESKD) occurring at a median age of 24–34 years [4–6]. Both nephrocalcinosis (NC) and kidney stones (KS) are common features of PH1, but are pathophysiologically distinct entities [7]. In PH1 patients, NC describes calcium oxalate (CaOx) crystal deposition within in renal tubular cells, interstitium or tubular lumen, either at the corticomedullary juncture or within the medulla. NC does not always lead to KS formation and KS can occur in the apparent absence of NC, but these two also often occur together in the same patient. Thus although these 2 pathologies are distinct they are also intimately related, and have many common risk factors [7, 8]. An experimental animal studies suggest that NC involved NLRP3 (nucleotide-binding oligomerization domain (NOD)-leucine rich repeats (LLR)-and pyrin domain-containing protein 3) mediated-activation of pro-inflammatory and pro-fibrotic macrophages and suppression of anti-inflammatory macrophages [9]. KS on the other hand form in the renal calyx attached to the papillae via tubular plugs or interstial Randall’s plaque. The vast majority (> 95%) of KS in PH patients are composed of CaOx monohydrate [10]. Although KS cause much morbidity and expense, NC is perhaps more ominous since it is associated with CKD risk [7]. Thus a key gap in knowledge is the precise basic renal cellular mechanisms involved in NC and KS formation, and the specific renal cellular and protein biomarkers of key pathophysiological processes that occur during renal calcification.

Kidney cells can be injured by increased local concentrations of oxalate or by CaOx crystals [11, 12]. Some studies support a role for renal tubular cell injury in the pathophysiology of nephrolithiasis, especially that associated with hyperoxaluria [11, 13, 14]. Such activated or injured cells could release biologically active membrane-bound extracellular vesicles (EVs; microvesicles (40–1000 nm) from the plasma membrane and exosomes (30–150 nm) from mature endosome) that can reflect and/or mediate early and late disease process [15–17]. Indeed, our previous studies demonstrated a relationship between specific populations of urinary EVs and KS disease, revealing a different urinary EV pattern between first time (incident) stone formers, prevalent calcium stone formers undergoing surgical procedures, and age- and sex-matched non-stone formers [17, 18].

A detailed urinary biomarker profile including EV characterization that could possibly elucidate potential mechanisms of KS and NC in PH1 has not yet been reported. Specific renal tubular and interstitial inflammatory cellular biomarkers that reflect early as well as late disease processes in PH1 patients are needed to identify specific targets or biomarkers for therapeutic interventional trials. We hypothesized that PH1 patients with ongoing intrarenal pathological calcification (NC or KS) excrete distinct populations of EVs containing specific renal and interstitial cellular, inflammatory and injury markers into their urine due to localized renal cellular events. Furthermore, those EVs that are present in the urine of PH1 patients may contain unique renal tubular and interstitial cell injury and inflammatory biomarkers that could be used to differentiate NC and KS pathologies, and to monitor these disease activities. To test this hypothesis, urinary EV populations and candidate soluble proteins involved in soft tissue calcifications were measured in PH1 patients with and without NC or KS.

## Results

### Clinical characteristics

In general, the age, body mass index, systolic and diastolic blood pressures, serum creatinine, eGFR, and 24 h urine volume, urine pH, and excretions of calcium, creatinine, and total protein were comparable in PH1 patients without and with NC or KS (Table 1). Urine citrate excretion trended lower in PH1 patients with KS compared to other groups. Urine osmolality tended lower whereas urinary oxalate trended higher in PH1 patients with NC and KS relative to patients without NC or KS (Table 1).

**Table 1:** Baseline clinical characteristics of study patients

Clinical characteristics	Primary hyperoxaluria type 1 (PH1)				
	PH1 without NC or KS (n = 10)	PH1 with NC (n = 6)	PH1 with KS (n = 9)	Total (n = 25)	*p* value
Age (years)	21 (18, 25)	16 (16, 17)	20 (17, 24)	18 (16, 22)	0.13
Sex					
Female (%)	7 (70%)	3 (50%)	2 (22%)	12 (48%)	0.14
Male (%)	3 (30%)	3 (50%)	7 (78%)	13 (12%)	
Body mass index (kg/m^2^)	24 (20, 33)	24 (23, 27)	24 (22, 28)	24 (22, 29)	0.94
Systolic blood pressure (mmHg)	111 (106, 116)	112 (100, 120)	112 (105, 118)	112 (105, 118)	0.84
Diastolic blood pressure (mmHg)	73 (67, 74)	73 (51, 74)	65 (56, 68)	68 (58, 74)	0.26
Serum creatinine (mg/dL)	0.9 (0.9, 1.2)	1.1 (0.8, 1.3)	1.3 (1.1, 1.4)	1.2 (0.9, 1.3)	0.16
eGFR (mL/min/1.73 m^2^)	79 (73, 91)	66 (55, 94)	68 (53, 76)	73 (55, 91)	0.66
*Urine biochemistry*					
Urine volume (mL/24 h)	2944 (2415, 3388)	3819 (3228, 5766)	2880 (2423, 3510)	3037 (2423, 3774)	0.19
pH	6.2 (6.0, 6.6)	6.6 (6.4, 6.8)	6.3 (6.2, 6.7)	6.4 (6.2, 6.7)	0.32
Osmolality (mOsm/kg)	610 (441, 632)	286 (237, 336)	316 (183, 516)	355 (248, 610)	0.06
Calcium (mg/24 h)	96 (67, 118)	90 (47, 148)	113 (59, 126)	105 (59, 130)	0.98
Citrate (mg/24 h)	482 (273, 537)	416 (386, 533)	300 (168, 467)	384 (249, 517)	0.53
Creatinine (mg/mL)	0.52 (0.32, 0.83)	0.43 (0.28, 0.44)	0.58 (0.50, 0.71)	0.50 (0.31, 0.71)	0.59
Total protein (mg/dL)	3.5 (0.32, 7.7)	10.0 (2.5, 17.0)	1.0 (1.0, 3.0)	3.0 (1.0, 9.0)	0.29
Urine oxalate (mmol/24 h/1.73 m^2^)	0.60 (0.35, 1.0)	1.0 (0.8, 1.7)	1.0 (0.6, 1.4)	0.8 (0.6, 1.5)	0.32

Data are presented as median (25th and 75th percentile). There were no statistically significant differences in parameters among groups (unadjusted *p* > 0.05)

### Urinary extracellular vesicles (EVs)

The total number of EVs and EVs from different types of glomerular and renal tubular cells (11/13 markers) were significantly (Q < 0.20) different between PH1 patients without NC and KS, and patients with NC or patients with KS (Table 2). Overall, PH1 patients with KS secreted fewer (median) urinary EVs of all types. The total number of phosphatidylserine (PS, annexin-V binding for most microvesicles)-and CD63 (exosome)-carrying EVs, EVs from glomerular cells (juxtaglomerular cells, podocytes) and cells of the proximal tubule, thick loop of Henle, distal tubule, collecting duct, and renal pelvis significantly differed between PH1 patients without NC or KS and patients with NC or KS (Table 2). Cellular adhesion/inflammatory (ICAM-1, MCP-1, and tissue factor) marker-carrying urinary EVs were statistically (Q < 0.20) different between PH1 patients without NC or KS and patients with NC or KS (Table 3). The number of VCAM-1 carrying EVs did not differ between groups (Table 3). Renal injury (β2-microglobulin, laminin α5, and NGAL) marker-positive urinary EVs were statistically (Q < 0.20) different between PH1 patients without and with NC or KS, whereas the number of clusterin and kidney injury molecule-1 (KIM-1)-carrying urinary EV did not differ between groups (Table 3). The urinary excretion of activated immune/inflammatory cell-derived EVs derived from total leukocytes, neutrophils, B-lymphocytes, T-lymphocytes, monocytes, M1-macrophages, M2-macrophages, and plasma cells were significantly (Q < 0.20) different among PH1 patients with NC or KS and PH1 patients without NC or KS (Table 4). The number of EVs bearing markers of EV generation (ANO4/ anoctamin 4 and HIP1/ Huntington interacting protein 1) were statistically (Q < 0.20) different among PH1 patients without NC or KS and with NC or KS (Table 5). The number of renal calcium/phosphate regulation or calcifying matrix vesicles markers (klotho, PiT1, and PiT2) were statistically (Q < 0.20) different between PH1 groups (Table 5). The number of FGF23 carrying EVs excreted into the urine did not differ between patients with NC or KS and those without NC or KS (Table 5).

**Table 2:** Total number of urinary extracellular vesicles derived from cells of different segments of nephron and renal pelvis in primary hyperoxaluria type 1 patients without and with nephrocalcinosis (NC) or kidney stones (KS)

Urinary extracellular vesicles (EVs)/mg creatinine from specific type of cells	Markers	Primary Hyperoxaluria Type 1 (PH1)			Statistical Results	
		PH-1 without NC or KS (n = 10)	PH-1 with NC (n = 6)	PH-1 with KS (n = 9)	*p* value	Q value
Microvesicles/phosphatidylserine	Annexin-V	14.4 (13.8, 15.2)	13.4 (12.6, 13.9)	12.3 (11.5, 13.6)	0.13	0.16*
Exosomes	CD63	14.0 (13.6, 14.4)	13.1 (12.2, 14.1)	12.8 (12.0, 13.4)	0.14	0.16*
*Extracellular vesicles from glomerular cells*						
Juxtaglomerular cells	Beta-1 adrenergic receptor	14.7 (13.8, 14.8)	13.8 (13.2, 14.1)	12.8 (11.9, 13.8)	0.10	0.16*
Mesangial cells	SM22 alpha	14.0 (12.7, 14.7)	13 (12.5, 13.2)	12.7 (11.9, 13.0)	0.06	0.13*
Podocytes	Nephrin	13.7 (12.2, 14.3)	13.7 (12.5, 14.6)	12.0 (11.5, 12.7)	0.03	0.12*
Bowman’s capsule—parietal cells	Cytokeratin 8	13.9 (12.7, 14.7)	13.2 (12.4, 14.0)	12.2 (11.7, 13.5)	0.26	0.28
*Extracellular vesicles from different segments of nephron and renal pelvis*						
Proximal tubule—simple cuboidal epithelium	Urate-anion Exchanger 1	12.8 (12.1, 13.9)	11.9 (11.6, 14.2)	11.4 (10.2, 12.0)	0.01	0.11*
	Megalin	14.2 (13.0, 14.6)	12.9 (12.0, 14.4)	11.9 (11.6, 13.0)	0.04	0.12*
Thin loop of Henle – simple squamous epithelium	Urea Transporter (SLC14A2)	11.9 (11.4, 12.8)	11.3 (10.4, 13.3)	11.1 (10.6, 12.4)	0.64	0.64
Thick loop of Henle—simple cuboidal epithelium	Uromodulin	15.0 (14.2, 15.7)	14.1 (13.1, 15.0)	12.6 (11.9, 14.4)	0.05	0.12*
Distal tubule—simple cuboidal epithelium	Prominin-2	14.5 (14.0, 15.2)	13.8 (12.7, 14.5)	13.0 (12.7, 13.2)	0.03	0.12*
Collecting duct—principal cells	Aquaporin-2	14.5 (13.7, 14.8)	13.8 (13.1, 14.4)	12.6 (12.0, 13.6)	0.09	0.16*
Renal pelvis—transitional epithelium	Cytokeratin 20	13.9 (12.3, 14.9)	12.8 (11.4, 14.5)	11.6 (11.5, 13.4)	0.12	0.16*

Data are presented as median (25th and 75th percentile) of natural log of respective markers positive for urinary EVs/mg creatinine

^*^False discovery rate (FDR) Q-value < 0.20 among PH1 patients groups

**Table 3:** Number of urinary extracellular vesicles (EVs) carrying cellular adhesion/inflammatory and renal injury molecules from primary hyperoxaluria type 1 patients without and with nephrocalcinosis (NC) or kidney stones (KS)

Urinary EVs/mg creatinine	Markers	Primary Hyperoxaluria Type 1 (PH1)			Statistical Results	
		PH1 without NC or KS (n = 10)	PH1 with NC (n = 6)	PH1 with KS (n = 9)	*p* value	Q value
Cellular adhesion/inflammatory markers	ICAM-1	13.5 (12.0, 13.7)	12.2 (11.7, 12.7)	11.4 (11.0, 12.0)	0.02	0.13*
	MCP-1	12.9 (12.3, 14.0)	12.5 (12.0, 12.8)	11.5 (11.3, 13.0)	0.13	0.17*
	Tissue factor	13.9 (13.2, 14.8)	13.4 (12.4, 14.50)	12.3 (12.2, 12.4)	0.02	0.13*
	VCAM-1	11.5 (10.0, 12.2)	10.7 (9.9, 11.0)	11.0 (10.2, 11.1)	0.73	0.73
Renal cellular injury markers	β2-microglobulin	13.2 (12.8, 13.9)	12.8 (12.5, 13.5)	11.6 (11.5, 12.6)	0.12	0.17*
	Clusterin	14.0 (13.4, 14.6)	13.0 (11.8, 14.1)	12.7 (11.8, 13.7)	0.19	0.23
	KIM-1	12.9 (11.5, 14.1)	11.0 (10.2, 12.5)	11.3 (10.9, 13.0)	0.22	0.25
	Laminin α5	14.0 (13.1, 14.2)	13.3 (12.5, 13.6)	12.6 (12.0, 13.0)	0.09	0.16*
	NGAL	14.2 (13.1, 15.0)	12.7 (11.9, 13.7)	12.2 (11.6, 13.7)	0.07	0.15*

Data are presented as median (25th and 75th percentile) of natural log of respective markers positive for urinary EVs/mg creatinine

^*^False discovery rate (FDR) Q-value < 0.20 among PH1 patients groups

**Table 4:** Number of urinary extracellular vesicles (EVs) derived from activated immune/inflammatory cells of primary hyperoxaluria type 1 patients without and with nephrocalcinosis (NC) or kidney stones (KS)

Urinary EVs/mg creatinine	Markers	Primary Hyperoxaluria Type 1 (PH1)			Statistical Results	
		PH1 without NC or KS (n = 10)	PH1 with NC (n = 6)	PH1 with KS (n = 9)	*p* value	Q value
Total leukocyte-derived	CD45	11.3 (11.1, 11.5)	11.3 (11.1, 12.7)	10.2 (10.0, 10.9)	0.03	0.13*
Neutrophil-derived	CD15	11.9 (11.7, 12.5)	11.8 (11.1, 13.4)	10.6 (10.2, 11.8)	0.07	0.15*
B-lymphocyte-derived	CD19	11.4 (10.9, 12.2)	11.7 (10.7, 12.9)	9.8 (9.5, 10.9)	0.04	0.13*
T-lymphocyte-derived	CD3	11.2 (11.0, 11.5)	11.0 (10.8, 11.2)	10.1 (9.7, 10.8)	0.07	0.15*
Monocyte-derived	CD14	11.6 (10.7, 12.4)	12.6 (11.5, 12.9)	10.3 (10.0, 11.5)	0.03	0.13*
M1-macrophage-derived	CD68	11.7 (10.9, 11.9)	12.1 (11.1, 12.4)	10.5 (10.0, 11.0)	0.03	0.13*
M2-macrophage-derived	CD206	11.2 (10.8, 12.2)	11.2 (10.3, 12.4)	10.2 (9.2, 10.5)	0.11	0.17*
Plasma cell-derived	CD138 + CD319	10.7 (10.4, 11.8)	11.8 (10.6, 13.5)	10.2 (9.3, 10.9)	0.05	0.14*

Data are presented as median (25th and 75th percentile) of natural log of respective markers positive for urinary EVs/mg creatinine

^*^False discovery rate (FDR) Q-value < 0.20 among PH1 patients groups

**Table 5:** Number of urinary extracellular vesicles (EVs) positive for EV generation, renal calcium and phosphate homeostasis regulators biomarkers from primary hyperoxaluria type 1 patients without and with nephrocalcinosis (NC) or kidney stones (KS)

Urinary EVs/mg creatinine	Markers	Primary Hyperoxaluria Type 1 (PH1)			Statistical Results	
		PH1 without NC or KS (n = 10)	PH1 with NC (n = 6)	PH1 with KS (n = 9)	*p* value	Q value
EV generation from plasma membrane	Anoctamin 4	12.6 (11.9, 13.8)	13.0 (11.7, 14.5)	11.3 (11.0, 11.6)	0.05	0.13*
Endocytosis mediated EV generation	Huntington interacting protein 1	11.9 (10.6, 13.2)	11.5 (10.7, 14.1)	10.2 (9.9, 10.8)	0.14	0.17*
Renal calcium/phosphate homeostasis regulators	Klotho	13.0 (12.5, 13.9)	12.3 (12.1, 14.2)	11.6 (10.3, 12.0)	0.04	0.13*
	Fibroblast growth factor 23	11.4 (11.1, 11.7)	10.9 (10.5, 11.2)	10.6 (10.2, 10.9)	0.26	0.28
	Phosphate transporter 1 (PiT1)	11.8 (11.5, 12.7)	11.6 (10.9, 12.1)	11.0 (10.3, 11.2)	0.15	0.19*
	Phosphate transporter 2 (PiT1)	12.3 (11.5, 13.5)	12.4 (11.5, 14.2)	11.2 (10.8, 12.4)	0.11	0.17*

Data are presented as median (25th and 75th percentile) of natural log of respective markers positive for urinary EVs/mg creatinine

^*^False discovery rate (FDR) Q-value < 0.20 among PH1 patients groups

### Urinary proteins measured by customized antibody arrays

The urinary concentration of proteins detected by a customized antibody array membrane designed for soft tissue calcification proteins is shown in Table 6 and Additional file 1: Figure 1. Many urinary proteins were detected by antibody array and 13 of total 40 proteins were significantly (Q < 0.20) different between PH1 patients without NC and KS and with NC or KS (Table 6). Significantly different by patient group were CD14, CD40, coagulation factor VII, C-reactive protein, E-cadherin, epidermal growth factor, endoglin, fetuin A, monocyte chemoattractant protein-1, neprilysin, osteopondin, osteoprotegrin, and platelet derived growth factor receptor beta (Table 6). Urinary excretion of other proteins did not differ among patient groups (Table 6).

**Table 6:** Expression of selected urinary proteins between PH1 patients without and with nephrocalcinosis (NC) or kidney stones (KS)

Measured urinary proteins by antibody array	Primary hyperoxaluria type 1 (PH1)			Statistical Results	
	PH1 without NC or KS (n = 6)	PH1 patients with NC (n = 6)	PH1 patients with KS (n = 6)	*p* value	Q value
Complement C5a	1.0 (0.9, 1.9)	1.0 (0.7, 1.4)	2.0 (1.2, 2.7)	0.08	0.23
CD14	12.8 (6.1, 15.9)	4.5 (3.7, 5.6)	7.1 (5.1, 9.6)	0.02	0.12*
CD40	2.3 (1.6, 4.1)	1.6 (1.3, 2.0)	3.4 (2.1, 4.5)	0.00	0.12*
Coagulation factor III	0.8 (0.4, 2.0)	0.6 (0.4, 1.0)	1.1 (0.5, 1.9)	0.42	0.56
Coagulation factor VII	2.8 (2.5, 4.3)	1.9 (1.6, 3.1)	3.6 (2.5, 5.0)	0.06	0.19*
Coagulation factor XIV	2.3 (1.5, 3.8)	1.6 (1.5, 2.7)	3.0 (1.8, 4.3)	0.28	0.43
C-reactive protein	18.9 (15.0, 24.0)	13.9 (12.0, 15.5)	16.9 (14.4, 20.7)	0.01	0.12*
E-cadherin	3.7 (2.0, 5.8)	2.1 (1.8, 2.6)	3.7 (3.0, 4.6)	0.03	0.14*
EGFR	0.7 (0.4, 1.6)	0.5 (0.3, 1.0)	1.2 (0.9, 1.5)	0.04	0.16*
Endoglin	10.7 (9.1, 14.6)	9.8 (7.9, 9.9)	13.0 (11.8, 16.1)	0.02	0.12*
E-selectin	16.0 (14.9, 18.2)	13.8 (12.3, 16.9)	17.4 (16.1, 19.0)	0.27	0.42
Fetuin A	72.6 (45.7, 138.5)	31.8 (9.9, 56.4)	70.5 (40.5, 97.4)	0.02	0.12*
Glycoprotein VI	3.5 (1.9, 7.4)	3.0 (2.0, 3.6)	4.9 (2.6, 8.1)	0.42	0.56
ICAM-1	13.2 (8.4, 18.2)	9.9 (8.4, 13.1)	16.4 (11.9, 20.8)	0.09	0.25
Interleukin 10	0.7 (0.4, 1.1)	0.5 (0.3, 0.8)	0.8 (0.4, 1.3)	0.13	0.28
Insulin receptor	3.7 (3.2, 7.0)	3.5 (2.9, 3.8)	4.8 (2.6, 8.0)	0.26	0.42
LDLR	5.0 (4.3, 7.8)	4.2 (3.3, 4.4)	6.3 (3.2, 10.6)	0.16	0.30
MCP-1	27.0 (19.6, 32.9)	15.7 (11.8, 18.4)	23.9 (18.6, 30.6)	0.01	0.12*
MMP-2	4.0 (3.4, 9.9)	3.3 (2.8, 3.6)	4.7 (2.8, 7.1)	0.12	0.28
MMP-9	5.7 (4.3, 16.1)	3.8 (3.0, 5.2)	5.3 (2.9, 14.0)	0.38	0.54
Neprilysin	1.0 (0.8, 1.8)	0.7 (0.5, 0.9)	1.9 (1.1, 3.1)	0.02	0.12*
Osteopontin	50.8 (17.2, 80.1)	9.8 (7.5, 28.3)	46.5 (17.6, 74.5)	0.06	0.19*
Osteoprotegrin	9.5 (8.3, 12.8)	7.7 (6.5, 10.2)	11.0 (9.6, 15.4)	0.04	0.16*
PAI-1	0.8 (0.5, 1.1)	0.6 (0.3, 1.0)	0.9 (0.8, 1.0)	0.14	0.28
PDGFRβ	7.1 (5.3, 9.4)	5.3 (4.1, 6.6)	7.6 (5.8, 10.9)	0.06	0.19*
PDGF-AA	2.7 (1.3, 3.5)	2.2 (1.7, 2.9)	3.0 (1.4, 4.7)	0.71	0.77
PDGF-BB	6.1 (3.7, 8.7)	4.2 (3.1, 6.3)	8.2 (4.7, 11.9)	0.11	0.28
PECAM-1	4.5 (3.3, 6.2)	3.8 (3.2, 4.2)	4.4 (2.4, 6.2)	0.67	0.77
Pref-1	7.5 (3.6, 12.4)	5.4 (4.9, 8.2)	6.0 (3.0, 9.8)	0.71	0.77
P-selectin	16.6 (12.6, 19.6)	15.5 (13.6, 17.3)	15.9 (11.3, 21.3)	0.91	0.91
RANTES	13.5 (12.1, 24.8)	12.9 (11.8, 14.7)	15.1 (12.2, 18.6)	0.36	0.54
TFPI	1.3 (1.2, 2.9)	1.3 (1.2, 1.7)	2.1 (1.1, 3.1)	0.49	0.61
Thrombomodulin	1.8 (1.2, 2.9)	1.6 (1.2, 2.1)	2.4 (1.3, 3.2)	0.25	0.42
TIMP1	9.0 (4.7, 15.0)	4.7 (2.7, 7.2)	9.3 (3.8, 12.9)	0.14	0.28
TIMP2	4.9 (3.8, 9.7)	1.9 (1.1, 6.3)	3.7 (2.0, 9.1)	0.16	0.30
TLR4	1.2 (0.7, 1.8)	1.0 (0.6, 1.3)	1.2 (0.7, 1.5)	0.52	0.63
Troponin I	10.5 (7.5, 14.4)	10.5 (8.4, 11.2)	10.6 (5.4, 16.1)	0.91	0.91
VCAM-1	2.2 (1.3, 3.4)	2.2 (1.6, 2.8)	2.7 (1.7, 3.9)	0.45	0.58
VE-cadherin	0.8 (0.5, 1.4)	1.1 (0.7, 1.2)	0.9 (0.6, 1.6)	0.62	0.73
VEGFR1	7.4 (5.6, 10.4)	7.5 (5.2, 8.6)	7.6 (5.7, 11.1)	0.88	0.91

Data are presented as medians (25th and 75th percentile) of relative intensity of selected urinary proteins average pixel value

C5a, complement component 5a; CD14, cluster of differentiation 14; CD40, cluster of differentiation 40; E-cadherin, epithelial cadherin; EGFR, epidermal growth factor receptor; E-selectin, endothelial selectin; ICAM-1, intercellular adhesion molecule-1; LDLR, low density lipoprotein receptor; MCP-1, monocyte chemoattractant protein-1; MMP-2, matrix metalloproteinase-2; MMP-9, matrix metalloproteinase-9; PAI-1, plasminogen activator inhibitor-1; PDGFRβ, platelet-derived growth factor receptor beta; PDGF-AA, platelet-derived growth factor-AA; PDGF-BB, platelet-derived growth factor-BB; PECAM-1, platelet endothelial cell adhesion molecule-1; pref-1, preadipocyte factor-1; RANTES, regulated upon activation, normal T-cell expressed and secreted; TFPI, tissue factor pathway inhibitor; TIMP1, tissue inhibitor of matrix metalloproteinase 1; TIMP2, tissue inhibitor of matrix metalloproteinase 2; TLR4, toll like receptor 4; VCAM-1: vascular cell adhesion molecule-1; VE-cadherin, vascular endothelial cadherin; VEGFR1, vascular endothelial growth factor receptor 1

^*^False discovery rate (FDR) Q-value < 0.20 among PH1 patients groups

## Summary

**Fig. 1:**
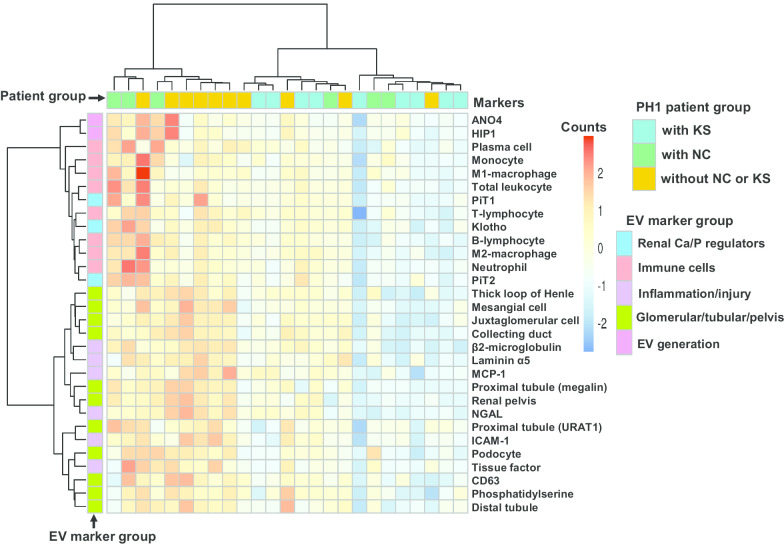
Biclustered heat map of significantly (Q < 0.20) changed biomarker carrying urinary EVs are presented as biomarkers (rows) by samples (columns). Urinary EVs counts are presented as feature-scaled values. PH1 subgroup is illustrated using column side colors and EV marker group is illustrated by row side colors

**Fig. 2:**
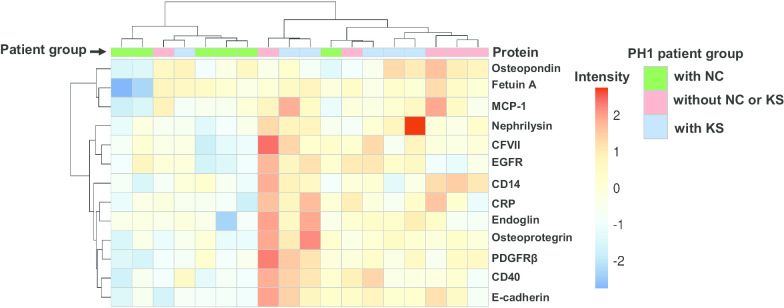
Biclustered heat map of significantly (Q < 0.20) altered urinary proteins are presented as biomarkers (rows) by samples (columns). Intensities are presented as feature-scaled values. PH1 subgroup is illustrated using column side colors

**Fig. 3:**
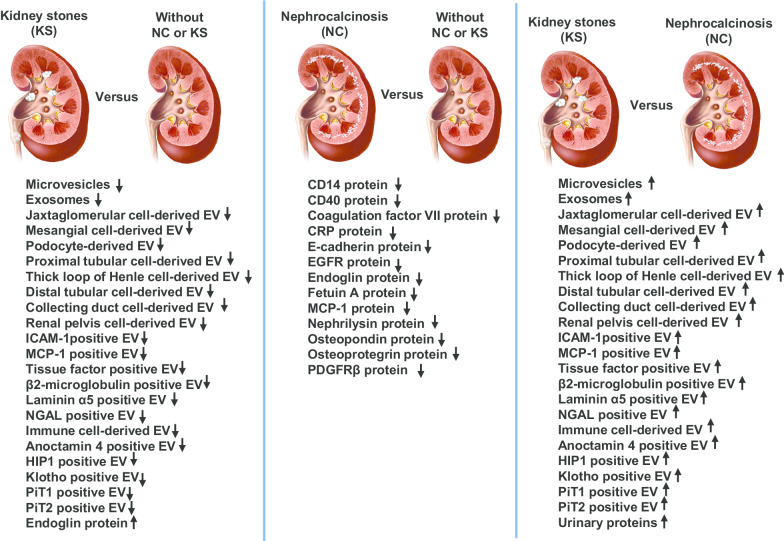
Summary of statistically (Q < 0.20) changed urinary EVs and proteins biomarkers median values from data Tables 2, 3, 4, 5 and 6 of each patients group compared to each other groups. Downward arrow indicates decreased whereas upward arrow indicates increased compared to respective group

We observed statistically significant differences between PH1 patients without NC and KS and those with NC or KS for 30/36 (80%) urinary EV biomarkers (Tables 2, 3, 4 and 5) and 13/40 (33%) urinary protein biomarkers (Table 6). Bicluster heat maps were used to display the urinary EV (Fig. 1) and protein (Fig. 2) biomarkers that differed (Q < 0.20) between groups. Figure 3 provides overall summary of the urinary EV and protein markers that differed by group.

## Discussion

PH1 can present as early as infancy to the sixth decade of life, and if not diagnosed early and treated appropriately can result in significant morbidity and mortality. Progressive CKD is common, with ESKD often resulting by the 6th decade [6]. NC and KS are common features of PH1 and have many common risk factors including hypercalciuria, hypocitriuria, but are pathophysiologically different entities [7, 8, 19]. Urine osmolality tended to be lower in PH1 patients with NC or KS compared to those without NC or KS. Previous studies have also reported decreased urine osmolality in patients with NC and/or KS [20, 21]. Since a lower urine concentration should protect against rather than favor crystallization, these changes are likely a result rather than cause of NC.

Currently no biomarkers have been identified that can differentiate between these two disease processes or underlying pathogenic mechanisms. The major observation in the present study is that PH1 patients with KS excrete significantly fewer EVs of most types compared to PH1 patients without NC or KS (Figs. 1 and 3). In contrast, PH1 patients with KS had a significantly greater urinary concentration of specific calcification-related proteins compared to patients with NC (Figs. 2 and 3). Although most urinary EV populations did not differ between PH1 patients with NC and KS, the urinary concentration of certain calcification-related proteins did. The distinct patterns of urinary EVs and proteins observed in PH1 patients without and with NC or KS suggest that these biomarkers reflect NC or KS status, and further validation of urinary EVs and proteins in larger patient populations may allow identification of novel biomarkers and provide clues regarding the pathogenesis of renal calcification in these diseases.

Phosphatidylserine (PS) expression on the surface of urinary EVs was determined by annexin-V binding. Normally PS is present in the inner membrane of asymmetric or non-activated cells. It has long been proposed that outer-membrane exposure of negatively charged PS in response to an extracellular fluid containing a high concentration of calcium, often after cellular activation or injury, is an important step during dystrophic calcification due to the high affinity of calcium for PS [13, 22, 23]. Most (> 80–95%) blood and urinary EVs carry surface PS [17, 18, 24–26]. In fact, an abundance of membranous vesicles and other unidentified organic matrices were observed in decalcified urinary stones formed in vitro and in vivo [22, 27–31]. The reduced urinary excretion of EVs (microvesicles and exosomes) observed in the PH1 patients with KS compared PH1 patients without NC or KS suggest they may be trapped within KS or taken up into nascent crystals in tubular fluid. Thus, it is not clear if the reduced number of EVs reflects enhanced ongoing crystallization, or in some way contributed to it.

The renal distribution of infiltrating inflammatory/immune cells are influenced by the composition of the glomerular filtrate and tubular fluid as well as ongoing pathological processes within the kidney [32]. Experimental animal studies have demonstrated that CaOx crystals that form in the lumen can transcytose into the interstitium and attract inflammatory cells including leukocytes, lymphocytes, monocytes and macrophages [33]. These cells could contribute to renal damage by secreting proteolytic enzymes, cytokines and chemokines [34–37], but the mechanisms by which inflammatory cells enter the interstitium are not known. A greater urinary excretion of oxalate ions and formation of CaOx microcrystals could activate renal cells to express/secrete chemoattractant and cell adhesion molecules to recruit inflammatory cells. Interstitial crystals are often surrounded by inflammatory cells including macrophages [37–39]. Our recent study demonstrated that the total number of urinary EVs carrying MCP-1 and NGAL derived from proximal nephron, thin descending limb, and papillary duct cells were significantly lower in idiopathic first time calcium stone formers compared to age-/sex-matched controls [18]. Intrarenal crystal deposition is a common pathway to induce kidney inflammation and injury [37, 40]. Phagocytes recruited to the interstitium could contribute to crystal integration and clearance [37]. Indeed, immunostaining and transmission electron microscopy studies demonstrated that interstitial macrophages ingest crystals and form multinucleated giant cells around larger sized crystals [39, 41, 42]. In the rodent kidney, macrophages can metabolize internalized CaOx crystals [39]. Subsequent events include take up into lysosomes and leak of lysosomal contents to activate NLRP3 inflammasome stimulated caspase-1 activation mediated secretion of IL-1β and IL-18 that recruit inflammatory cells to the site of crystal formation. In the current study the lower number of EVs carrying inflammatory molecules (Figs. 1 and 3 and Table 3) in PH1 patients with KS compared PH1 patients without NC or KS could potentially reflect dysregulation of this process. The significantly different urinary EV populations derived from activated immune/inflammatory cells observed between PH1 patients with KS versus those with NC (Figs. 1 and 3 and Table 4) are consistent with the distinct pathogenic processes between these two diseases.

ANO4 is a calcium-dependent non-selective cation channel involved in Ca^++^ regulation within the endoplasmic reticulum (ER) and Ca^++^ dependent plasma membrane lipid scramblase activity [43, 44]. Expression of ANO4 attenuates Ca^++^ leakage from the ER [44]. Cell membrane phospholipid asymmetry can result from intracytoplasmic Ca^++^ activation of lipid scramblase that inhibits aminophospholipid translocase activity [45]. This process results in redistribution and stable expression of negatively-charged PS to the outer membrane and the production of microvesicles [45]. HIP1 is one of the proteins that regulate clathrin assembly during endocytosis [46, 47], and it is expressed in mouse and human glomerular podocytes [48]. Disruption of HIP1 was associated with decreased endocytosis-mediated receptor trafficking in the central nervous system [49]. In the current study, the reduced number of ANO4- and HIP1-expressing urinary EVs observed in PH1 patients with KS compared PH1 patients without NC or KS and with NC may reflect changes in ER Ca^++^-mediated microvesicle production and HIP-mediated endocytic processes that promote exosome release. These in turn may reflect dysregulation in endocytic processes integral for the prevention of pathologic calcifications like KS.

Disturbances of renal Ca^++^ and phosphate (P_i_) homeostasis are linked to chronic renal insufficiency and urinary stone disease. Studies suggest that Ca^++^ and P_i_ homeostasis is regulated by klotho (type1 transmembrane protein) and FGF23 (paracrine/endocrine peptide). Membrane-bound klotho acts as a co-receptor for FGF23, and is abundantly expressed in the distal nephron and is also present in the proximal tubule lumen where it serves to inhibit Pi excretion by modulating Na-coupled Pi transporters [50]. FGF23 is a principal regulator of serum phosphorus concentration through α-klotho associated FGF receptor. The sodium phosphate cotransporters, PiT1 and PiT2, are ubiquitously expressed throughout the body including within the kidneys, and contribute to cellular phosphate uptake, maintenance of cellular phosphate hemostasis, regulation of proliferation/apoptosis, biomineralization, pathological calcification, and inflammation via matrix vesicles [51–55]. Matrix vesicles are a form of EVs derived from the plasma membrane of activated cells that possess membrane transporters and move calcium and phosphate from inside to outside the vesicles [56]. Matrix vesicles potentiate a microenvironment for calcium phosphate nucleation and biomineralization [56–59]. The lower number of urinary EVs expressing the calcium/phosphate regulators klotho, PiT and PiT2 in patients with KS compared patients without NC and KS (Figs. 1 and 3 and Table 5) may suggest that EVs contribute to stone formation and serve as crystal nucleation sites within the kidney.

Although many proteins have been implicated in crystal-renal cell interactions and KS pathogenesis [60–62], the exact group of proteins ultimately involved in soft tissue calcification are not completely known. At a minimum, the differential expression of urinary proteins in PH1 patients with NC compared to PH1 patients without NC or KS and with KS suggests NC is different from stone pathogenesis. In general a subset of proteins in our array was upregulated in the KS population and downregulated in the NC group (Figs. 2 and 3 and Table 6). Those upregulated in the KS group might reflect ongoing subtle inflammation (e.g., CD14, CD40, Endoglin) and injury/matrix remodeling (e.g., Osteopontin, E-selectin, EGFR, PDGFRβ), as has been suggested by others [60–66]. Downregulation in the NC group (e.g., MCP-1 and OPN) might reflect loss of proximal tubular function and/or cell number, and ultimately contribute to CKD progression. These points need further validation in future studies with larger populations.

This study has some limitations. The sample size is small for each group due to rare nature of PH1. There is also a need to establish a standard method to express urinary EV data. In general, accounting for the variability in final urine concentration over time and between persons is an issue for all urinary biomarkers, including EVs. For urinary biomarkers that are filtered from blood, normalization to creatinine is often helpful to account for the variable concentration. For biomarkers that are secreted along the nephron, the utility of this approach can be variable. Thus many studies often analyze and report urine biomarkers as both raw concentrations as well as creatinine-normalized values. A recent study used cytosolic tumor susceptibility gene 101 protein for small (< 200 nm) EVs normalization [67] but there is no single surface marker established for urinary EV normalization, and the optimal approach to express urinary EV data is not yet established. The expression of the results as a percentage of positive EVs compared to the total number of EVs analyzed would be more appropriate approach for purified isolated EVs (via antibodies or sucrose density gradient based methods) but is not a suitable approach for cells-free whole urine due to the presence of similar size protein or chemical aggregates or particles that could confound this approach. The inflammatory cells and proteins biomarkers expressing EVs may not be solely from renal parenchyma and may also arise from other parts of the urological system and filtered from blood. Future studies will be necessary that employ combinations of inflammatory markers and specific renal cell specific markers to resolve this issue in the future [18].

## Conclusions

This study demonstrated that PH1 patients with KS excreted fewer EVs, while the urinary concentration of specific calcification-related proteins were greater in PH1 patients with KS compared to PH1 patients without NC or KS or with NC. The distinct urinary EV populations and expression of specific type of proteins observed between PH1 patients without and with NC or KS suggest that specific populations of urinary EVs and proteins reflect NC or KS status. Further analysis of these urinary EVs biomarkers and their bioactive (DNA, RNAs, proteins, and metabolites) molecules and pathway analysis of differentially expressed urinary proteins in larger patient population may provide potential biomarkers to elucidate the pathogenic mechanisms of KS in PH1 patients with treatments options for these adverse pathogenic events.

## Methods

### Chemicals, reagents, and antibodies

Recombinant annexin-V protein and mouse anti-human cluster of differentiation 3 (CD3), CD14, CD15, CD19, CD45, CD54, CD63, CD68, CD106, CD138, CD163, and CD206 antibodies conjugated with fluorescein isothiocyanate (FITC) or R-phycoerythrin (PE) and TruCOUNT™ (4.2 µm) beads were purchased from BD Biosciences, San Jose, CA, USA. FITC conjugated mouse anti-human tissue factor was purchased from American Diagnostica Inc., Stamford, CT, USA. Fluorophore-conjugated mouse anti-human monocyte chemotactic protein-1 (MCP-1), mouse anti-human β2-microglobulin, mouse anti-human CD365 (kidney injury molecule-1), and mouse anti-human CD319 antibodies were purchased from BioLegend Inc., San Diego, CA, USA. Fluorophore-conjugated rabbit anti-human laminin α5, anti-human neutrophil gelatinase-associated lipocalin (NGAL), anti-human SM22-alpha, anti-human nephrin, anti-human cytokeratin 8, anti-human urate transporter 1, anti-human megalin, anti-human SLC14A2 (urea transporter), anti-human prominin 2, and anti-human cytokeratin 20 antibodies were obtained from Bioss Antibodies, Woburn, MA, USA. PE conjugated mouse anti-human clusterin antibody purchased from Novus Biologicals, LLC, Centennial, CO, USA. FITC conjugated rabbit anti-human β1-adrenergic receptor, anti-human nephrin, anti-fibroblast growth factor 23 (FGF23), and anti-human aquaporin-2 antibodies were obtained from Biorbyt, Cambridge, Cambridgeshire, UK. PE conjugated rabbit anti-human Huntington interacting protein 1 (HIP1), anti-human SLC20A1 (PiT1, phosphate transporter 1), anti-human SLC20A2 (PiT2), and anti-human Klotho antibodies and FITC conjugated mouse anti-human uromodulin antibody were from Lifespan Biosciences, Inc. Seattle, WA, USA. FITC conjugated rabbit anti-human anoctamin-4 (ANO4) antibody was obtained from United States Biological, Salem, MA, USA. HEPES (4-(2-hydroxyethyl)-1-piperazineethanesulfonic acid), and Hanks’ balanced salts were purchased from Sigma Chemicals Co., St. Louis, MO, USA. All other reagents and solvents used in this study were of analytical/reagents grade.

### Study patients

This study was approved by the Institutional Review Board at Mayo Clinic, Rochester, MN, USA. PH1 study patients were enrolled in the Mayo Clinic Rare Kidney Stone Consortium (RKSC) PH Registry. Bio-banked cell-free 24 h urine samples were collected in toluene preservative from male (n = 13) and female (n = 12) PH1 patients aged from 15–30 years following informed consent for participation in the Rare Kidney Stone Consortium (RKSC) biobank. PH patients without (n = 10) and with NC (no KS; n = 6) or KS (no NC; n = 9) were identified from RKSC registry image data obtained during the time of sample collection for RKSC bio-banking. One patient with both KS and NC was not included in this study. The cohorts of PH patients were selected for NC alone, KS alone, or neither KS nor NC at the time of kidney imaging closest to the urine sample. Most of the urine samples were collected on the same day however gaps of 1–9 months occurred in 3 subjects. All PH1 patients were diagnosed and confirmed by genetic testing and/or liver biopsy. Estimated glomerular filtration rate (eGFR) was calculated using the Schwartz formula [68] for children < 18 years of age and Modification of Diet in Renal Disease study formula [69] for patients > 18 years of age. PH1 patients with eGFR < 40 mL/min/1.73 m^2^ and prior kidney and/or liver transplantation were excluded.

### Serum and urine biochemistry

All serum and urine biochemistry measurements were performed in the Mayo Clinic Renal Testing Laboratory, Rochester, MN, using standard protocols/methods as in previous studies [17, 18, 24, 70].

### Quantification of urinary extracellular vesicles (EVs)

A standardized and validated method of digital flow cytometer (FACSCanto™) was used to define EVs by size from ≥ 200 nm to ≤ 1000 nm and larger molecular weight proteins associated smaller size exosomes and/or membrane-derived vesicles and annexin-V-fluorescence for quantification of selected biomarker-positive urinary EVs [17, 18, 24, 70]. Briefly, prior to analysis by flow cytometer all buffers, antibodies and reagents used in this study were filtered twice using a 0.2 µm-sized membrane filter to eliminate similar size chemical particles and protein aggregates to reduce instrument noise. The total concentration of EVs was first quantified in all samples using fluorescein conjugated annexin-V (binds surface phosphatidylserine (PS) of EVs) and CD63 (binds tetraspanin/surface glycoprotein present in EVs) to optimize the appropriate sample volume and flow rate for urinary EVs analysis. A volume of cell-free urine ranging between 5 and 80 µL was used for quantifying populations of marker-positive EVs using fluorophore conjugated antibodies. All flow cytometer settings, filtration and dilutions of fluorophore conjugated antibodies and reagents were similar to those previously published studies from our group [17, 18, 24, 70]. The absolute number of urinary EVs were calculated as the number of urinary EVs per µL of urine and also normalized to urine creatinine concentration as similar to our previous studies [17, 18, 24, 70].

### Relative intensity of expression of urinary protein analysis by customized antibody array

Diluted cell-free urine (500 µL urine + 500 µL blocking buffer) from PH1 patients (n = 6/group) was used to identify urinary excretion of proteins by a customized antibody array membrane from Ray Biotech, Inc., Peachtree Corners, GA, USA using the standard protocol suggested by the manufacturer. The densities of urinary protein signals were quantified by positive dot blot analysis of UN-SCAN-IT gel™ analysis software (Silk Scientific Inc., Orem, UT, USA). Positive and negative controls were included on the membrane arrays (Additional file 1: Figure 1). The signal intensity of every protein measured in cell-free urine was normalized by subtracting the average background signal (negative control) and dividing by the average pixel value of the positive controls on each membrane. The intensity of each protein was expressed as the average of duplicate pixel values for that protein on a given membrane similar to our previous studies using serum and platelet lysate [71, 72].

### Sample size by assay

Urinary EV data from flow cytometric analyses are presented from male and female PH1 patients without (n = 10) and with NC (n = 6) or KS (n = 9), whereas urinary proteins measured by customized antibody array are presented from male and female PH1 patients without (n = 6) and with NC (n = 6) or KS (n = 6). All analyses were adjusted for sex due to an unequal distribution of male and female patients between groups, and control of type I error rate was addressed using the false discovery rate (FDR) < 0.20 due to our goal to explore potential differences. The absolute number of urinary EVs was normalized to 24 h urine creatinine concentration. As in our previous studies [17, 18, 24, 70], similar results were obtained when urinary EVs were analyzed as a concentration (per µL of urine) or normalized to urine creatinine (per mg creatinine).

### Data analysis

All quantitative data are presented as median, 25th and 75th percentile and nominal data as counts and percentages. Comparisons of clinical characteristics as well as serum and urine biochemistry across groups were assessed using Kruskal–Wallis tests for quantitative traits and chi-squared tests with resampling-based *p* values for nominal traits. Hypothesis testing for group differences in specific biomarker-positive urinary EVs, and urinary protein array data were performed using analysis of covariance (ANCOVA) adjusting for sex. Given limited sample size to verify analytical assumptions, omnibus F-test *p* values were derived via permutation (B = 5000 permutations) using the approach of Freedman and Lane [73]. As the overall goal was to explore potential differences, Type I error control was addressed using the false discovery rate (FDR) method of Benjamini and Hochberg [74] based on the omnibus testing *p* values. Differences among groups were considered statistically significant at false discovery rate (FDR) < 0.20, and *p* values and corresponding Q-values (adjusted *p* values derived from an optimized FDR) are reported for all comparisons. These were derived separately for the EVs and urinary protein array data. For respective subsets of significant biomarkers, biclustered heat maps were generated using feature-scaled log-transformed data, with hierarchical clustering dendrograms generated using Euclidean distance and the Ward clustering criterion. All analyses were performed using the statistical software R v3.6.2.

## Supplementary information

**Additional file 1: Figure 1.** Example customized antibodies or proteins arrays from 24 h cell-free urine collected from type 1 primary hyperoxaluria (PH1) patients without nephrocalcinosis (NC) or kidney stones (A) and with NC (B) or Stones (C). The proteins or antibodies array template (D) is customized based on the proteins involved soft tissue calcification.

## Data Availability

The data sets used and/or analyzed during the current study are not publically available but available from first and senior author on reasonable request.

## Abbreviations

CD: Cluster of differentiation
EVs: Extracellular vesicles
HEPES: 4-(2-Hydroxyethyl)-1-piperazineethanesulfonic acid
KS: Kidney stone
NC: Nephrocalcinosis
PiT1: Phosphate transporter 1
PiT2: Phosphate transporter 2
C5a: Complement component 5a
CD14: Cluster of differentiation 14
CD40: Cluster of differentiation 40
CFIII: Coagulation factor III
CFVII: Coagulation factor VII
CFXIV: Coagulation factor XIV(protein C)
CRP: C-reactive protein
E-cadherin: Epithelial cadherin
EGFR: Epidermal growth factor receptor
E-selectin: Endothelial selectin
GP VI: Glycoprotein VI
FDR: False discovery rate
ICAM-1: Intercellular adhesion molecule 1
IL-10: Interleukin 10
Insulin R: Insulin receptor
LDLR: Low density lipoprotein receptor
MCP-1: Monocyte chemoattractant protein-1
MMP-2: Matrix metalloproteinase-2
MMP-9: Matrix metalloproteinase-9
OPN: Osteopontin
OPG: Osteoprotegrin
PAI-1: Plasminogen activator inhibitor-1
PDGFRβ: Platelet-derived growth factor receptor beta
PDGF-AA: Platelet-derived growth factor-AA
PDGF-BB: Platelet-derived growth factor-BB
PECAM-1: Platelet endothelial cell adhesion molecule-1
pref-1: Preadipocyte factor-1
PS: Phosphatidylserine
RANTES: Regulated upon activation, normal T-cell expressed and secreted
TFPI: Tissue factor pathway inhibitor
TIMP1: Tissue inhibitor of matrix metalloproteinase 1
TIMP2: Tissue inhibitor of matrix metalloproteinase 2
TLR4: Toll like receptor 4
VCAM-1: Vascular cell adhesion protein-1
VE-cadherin: Vascular endothelial cadherin
VEGFR1: Vascular endothelial growth factor receptor 1
