# Correction to: Specific populations of urinary extracellular vesicles and proteins differentiate type 1 primary hyperoxaluria patients without and with nephrocalcinosis or kidney stones

**DOI:** 10.1186/s13023-020-01671-7

**Published:** 2021-02-18

**Authors:** Muthuvel Jayachandran, Stanislav V. Yuzhakov, Sanjay Kumar, Nicholas B. Larson, Felicity T. Enders, Dawn S. Milliner, Andrew D. Rule, John C. Lieske

**Affiliations:** 1 Division of Nephrology and Hypertension, College of Medicine and Science, Mayo Clinic, 200 First Street SW, Rochester, MN 55905 USA; 2 Division of Hematology Research, College of Medicine and Science, Mayo Clinic, 200 First Street SW, Rochester, MN 55905 USA; 3 Department of Physiology and Biomedical Engineering, College of Medicine and Science, Mayo Clinic, 200 First Street SW, Rochester, MN 55905 USA; 4 Biomedical Statistics and Bioinformatics, College of Medicine and Science, Mayo Clinic, 200 First Street SW, Rochester, MN 55905 USA; 5 Department of Laboratory Medicine and Pathology, College of Medicine and Science, Mayo Clinic, 200 First Street SW, Rochester, MN 55905 USA

## Correction to: *Orphanet J Rare Dis* (2020) 15:319. https://doi.org/10.1186/s13023-020-01607-1

The original publication of this article [1] contained an error in Fig. 3. In the last column of **Kidney stones (KS) versus Nephrocalcinosis (NC)** all arrows pointed upwards to indicate an increase.

However, as shown in the data from the tables: all arrows in **Kidney stones (KS) versus Nephrocalcinosis** should point downwards to indicate a decrease. The exception is **Urinary proteins,** this remains an increase.

The incorrect (Fig. 1) and correct (Fig. 2) versions of Fig. 3 are published in this correction article. The original article has been updated with the correct version.

**Fig. 1:**
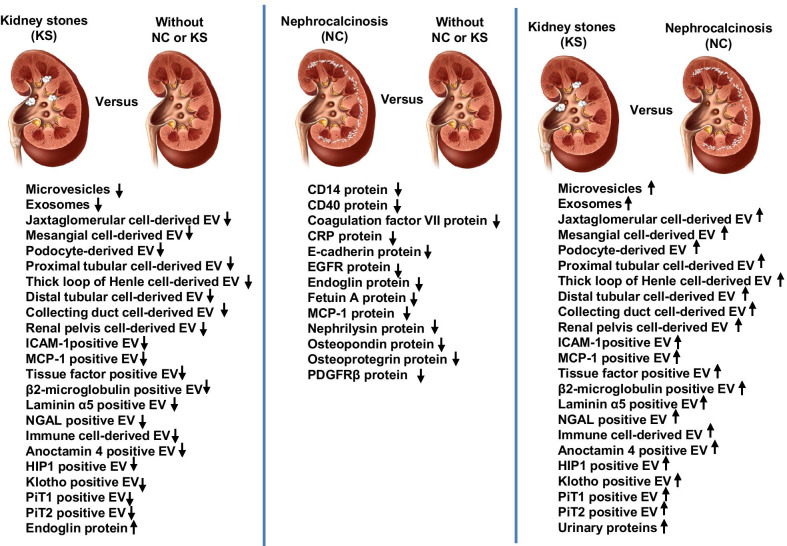
Incorrect version of Fig. 3 as originally published

**Fig. 2:**
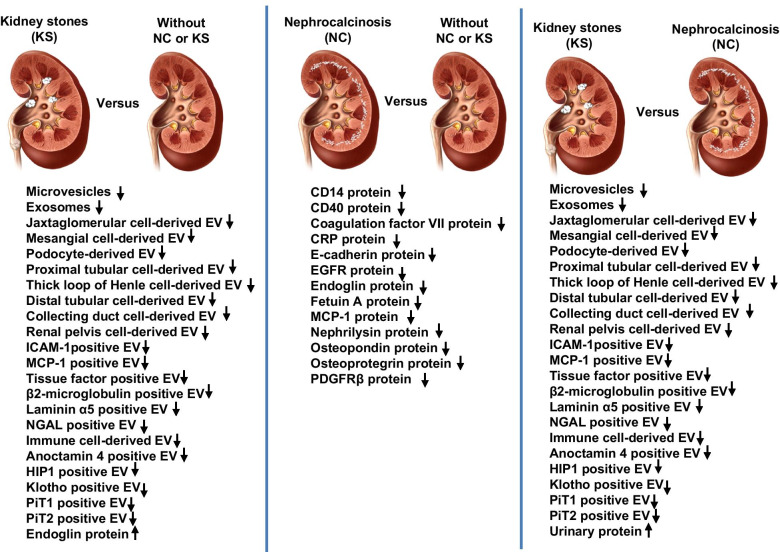
Correct version of Fig. 3 as corrected in the original publication
