# Supplementary material for: Specific populations of urinary extracellular vesicles and proteins differentiate type 1 primary hyperoxaluria patients without and with nephrocalcinosis or kidney stones
Source: Orphanet J Rare Dis. 2020 Nov 11;15:319. doi: 10.1186/s13023-020-01607-1 (PMC7659070; doi:10.1186/s13023-020-01607-1)
Supplement: Supplementary file 1 — Additional file 1: Figure 1. Example customized antibodies or proteins arrays from 24 h cell-free urine collected from type 1 primary hyperoxaluria (PH1) patients without nephrocalcinosis (NC) or kidney stones (A) and with NC (B) or Stones (C). The proteins or antibodies array template (D) is customized based on the proteins involved soft tissue calcification. [file 13023_2020_1607_MOESM1_ESM.pptx]

## Slide 1
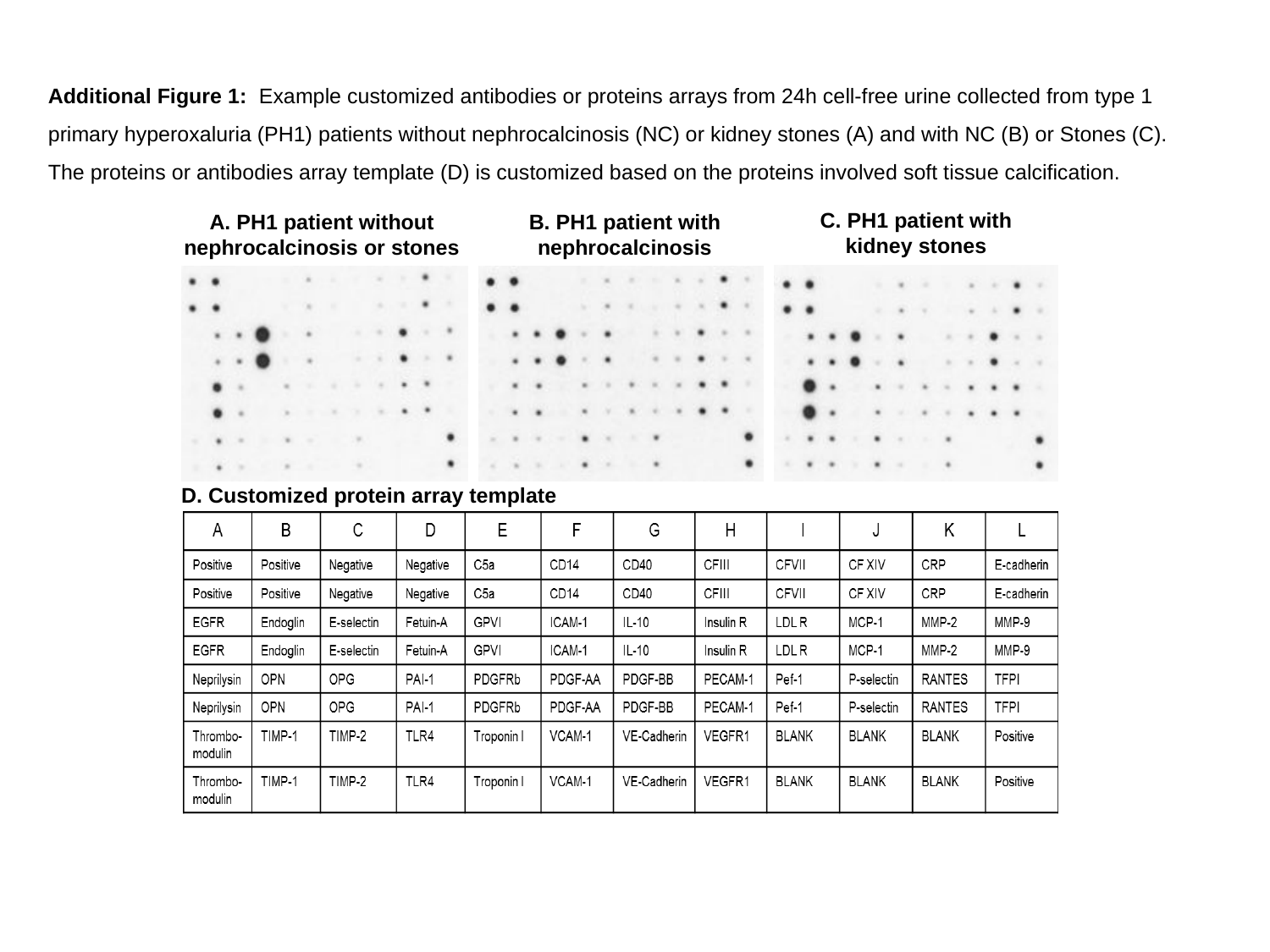

Additional Figure 1: Example customized antibodies or proteins arrays from 24h cell-free urine collected from type 1 primary hyperoxaluria (PH1) patients without nephrocalcinosis (NC) or kidney stones (A) and with NC (B) or Stones (C). The proteins or antibodies array template (D) is customized based on the proteins involved soft tissue calcification.
 C. PH1 patient with
kidney stones
 B. PH1 patient with
nephrocalcinosis
 A. PH1 patient without
nephrocalcinosis or stones
 D. Customized protein array template

## Slide 2
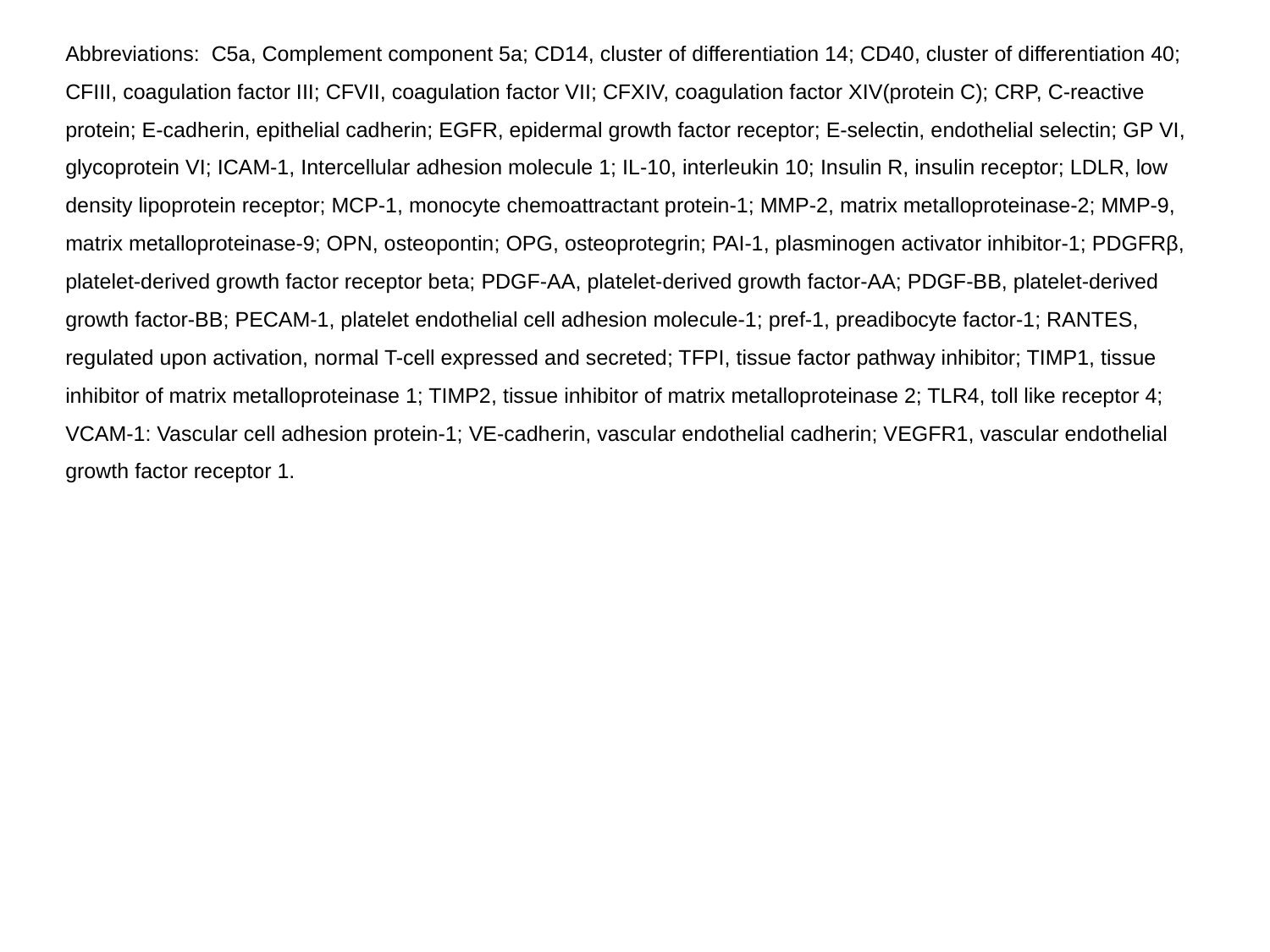

Abbreviations: C5a, Complement component 5a; CD14, cluster of differentiation 14; CD40, cluster of differentiation 40; CFIII, coagulation factor III; CFVII, coagulation factor VII; CFXIV, coagulation factor XIV(protein C); CRP, C-reactive protein; E-cadherin, epithelial cadherin; EGFR, epidermal growth factor receptor; E-selectin, endothelial selectin; GP VI, glycoprotein VI; ICAM-1, Intercellular adhesion molecule 1; IL-10, interleukin 10; Insulin R, insulin receptor; LDLR, low density lipoprotein receptor; MCP-1, monocyte chemoattractant protein-1; MMP-2, matrix metalloproteinase-2; MMP-9, matrix metalloproteinase-9; OPN, osteopontin; OPG, osteoprotegrin; PAI-1, plasminogen activator inhibitor-1; PDGFRβ, platelet-derived growth factor receptor beta; PDGF-AA, platelet-derived growth factor-AA; PDGF-BB, platelet-derived growth factor-BB; PECAM-1, platelet endothelial cell adhesion molecule-1; pref-1, preadibocyte factor-1; RANTES, regulated upon activation, normal T-cell expressed and secreted; TFPI, tissue factor pathway inhibitor; TIMP1, tissue inhibitor of matrix metalloproteinase 1; TIMP2, tissue inhibitor of matrix metalloproteinase 2; TLR4, toll like receptor 4; VCAM-1: Vascular cell adhesion protein-1; VE-cadherin, vascular endothelial cadherin; VEGFR1, vascular endothelial growth factor receptor 1.
